# Cicatriz Miocárdica e Repolarização Ventricular no Eletrocardiograma: Insights a Partir da Ressonância Magnética Cardíaca

**DOI:** 10.36660/abc.20210712

**Published:** 2021-10-06

**Authors:** Luciano C. Amado

**Affiliations:** 1 University of Minnesota Physicians MinneapolisMinnesota EUA University of Minnesota Physicians, Minneapolis, Minnesota – EUA

**Keywords:** Fibrose Endomiocárdica, Insuficiência Cardíaca, Volume Sistólico, Prognóstico, Morte Súbita Cardíaca, Eletrocardiografia/métodos, Espectroscopia de Ressonância Magnética/métodos

A fibrose miocárdica é uma condição comum final da maioria das vias inflamatórias, e é frequentemente vista em pacientes com insuficiência cardíaca e fração de ejeção reduzida (ICFEr).^1 , 2^ A presença de cicatriz miocárdica, detectada por ressonância magnética (RM) com contraste, tem se mostrado um preditor de prognóstico e morte súbita cardíaca em pacientes com ICFEr.^3 - 6^ A RM cardíaca (RMC) tem se destacado como um método não invasivo padrão-ouro para avaliação de cicatriz e predição de risco de arritmias ventriculares.^3 , 5 , 7 , 8^ Contudo, a RMC pode demandar tempo, ter custo elevado, e não ser facilmente disponível.

O eletrocardiograma (ECG) de 12 derivações é um instrumento diagnóstico simples e de baixo custo amplamente empregado na prática clínica para o diagnóstico de arritmias cardíacas. A forma de onda do ECG é a expressão dos potenciais de ação transmembrana e é composta por dois processos distintos: despolarização e repolarização.^9^ A repolarização ventricular é um fenômeno elétrico complexo que, na prática clínica é medida do início do complexo QRS ao final da onda T.^10^ Mudanças na estrutura e no sistema elétrico cardíacos podem causar anormalidades no potencial de ação, afetando o período refratário, favorecendo assim, o início das arritmias ventriculares.^10^

Nesta edição dos Arquivos Brasileiros de Cardiologia, Erturk et al.,^11^ realizaram um estudo observacional, retrospectivo, avaliando a relação entre a cicatriz miocárdica detectada por RM com contraste e vários parâmetros eletrocardiográficos de repolarização ventricular em pacientes com ICFEr. Os autores observaram medidas prolongadas do intervalo QTc, intervalo Tp-e e ângulo QRX-T frontal em pacientes com ICFEr que apresentaram cicatriz miocárdica na RM com contraste, em comparação a pacientes que não a apresentaram.

Com base na análise de múltiplas variáveis, o intervalo Tp-e foi o melhor parâmetro para predizer a presença de cicatriz miocárdica. Os autores concluíram que é possível predizer essa condição utilizando parâmetros eletrocardiográficos na ICFEr.^11^

Há poucos estudos sobre a relação entre a presença de cicatriz miocárdica e alterações no ECG. Modelos experimentais mostraram que a indução da formação de cicatriz subendocárdica prolonga o tempo de repolarização das células M,^9 , 12^ o que determina o intervalo QT. Estudos prévios mostraram uma forte associação entre presença de cicatriz subendocárdica e repolarização ventricular anormal em pacientes com doença arterial coronariana.^13^ Estudos com RM cardíaca de indivíduos da comunidade também demonstraram que a presença de cicatriz subclínica detectada por RM com contraste estava associada com maior mortalidade.^14^ Portanto, a repolarização ventricular anormal, detectada por ECG, pode ser útil na predição de cicatriz miocárdica subclínica e na identificação de pacientes em alto risco.

Apesar deste estudo apresentar resultados animadores que agregam importantes informações a publicações anteriores,^11 , 13 , 15^ o estudo apresenta limitações, incluindo o tamanho amostral pequeno, seleção de pacientes em um único centro, e delineamento retrospectivo e observacional. Serão necessários estudos grandes prospectivos para validar os parâmetros de repolarização ventricular propostos. Os autores estabeleceram uma base sólida de informações que promoverá o avanço da área de ECG em estudos futuros.

O estudo de Erturk et al.,^11^ também destaca a íntima relação entre fibrose miocárdica e distúrbios na condução elétrica. Existe um forte interesse em se compreender a composição da cicatriz e a instabilidade elétrica relacionada.^3 , 5 , 7^ Sabe-se que áreas de tecido cicatrizado exibem regiões de baixa condução que favorecem circuitos de reentrada e predispõem à taquicardia ventricular. No entanto, o mecanismo subjacente que conduz à fibrilação ventricular é menos claro. A instabilidade elétrica é raramente uma consequência de uma única anormalidade, e sim um processo dinâmico que envolve uma série de fatores que, em conjunto, conduzem a um evento fatal. Consequentemente, novos métodos incorporando diferentes modalidades são necessários para caracterizar a instabilidade elétrica ventricular. O presente estudo encoraja o desenvolvimento de estudos maiores que avaliem prospectivamente o uso de ECG para o rastreio de cicatriz miocárdica e consequente avaliação de risco de arritmia ventricular.
