# Supplementary material for: Cicatriz Miocárdica e Repolarização Ventricular no Eletrocardiograma: Insights a Partir da Ressonância Magnética Cardíaca
Source: Arq Bras Cardiol. 2021 Oct 6;117(4):688–9. [Article in Portuguese] doi: 10.36660/abc.20210712 (PMC8528371; doi:10.36660/abc.20210712)
Supplement: Translation

# Translation: Myocardial Scar and Ventricular Repolarization on the electrocardiogram: Insights from Cardiac Magnetic Resonance Imaging

**Authors:** Luciano C. Amado

**Affiliations:** 1 USA University of Minnesota Physicians, Minneapolis, Minnesota – USA

**Keywords:** Endomyocardial Fibrosis, Heart Failure, Stroke Volume, Prognosis, Death Sudden, Cardiac, Electrocardiography/methods, Magnetic Resonance Spectroscopy/methods

Myocardial fibrosis is a final common feature of most inflammatory pathway and is frequently seen in patients with heart failure with reduced ejection fraction (HFrEF).^1 , 2^ Myocardial scar, detected by contrast enhanced magnetic resonance imaging (ce-MRI), has been shown to be a predictor of prognosis and sudden cardiac death in patients with HFrEF.^3 - 6^ Cardiac MRI has emerged as a non-invasive gold standard for the assessment of scar and risk prediction of ventricular arrhythmias.^3 , 5 , 7 , 8^ Nevertheless, cardiac MRI can be time consuming, expensive, and not easily available.

The 12-lead surface electrocardiogram (ECG) is a simple and inexpensive diagnostic tool that is widely used in clinical practice for the diagnosis of cardiac arrhythmias. The ECG waveform is the expression of the transmembrane action potentials and is composed of two distinct processes: depolarization and repolarization.^9^ Ventricular repolarization is a complex electrical phenomenon which in clinical practice is measured from the beginning of the QRS complex to the end of the T-wave.^10^ Cardiac structural and electrical changes may cause action potential abnormalities, affecting the refractory period and thus favoring the onset of ventricular arrhythmias.^10^

In this issue of the *Arquivos Brasileiros de Cardiologia* , Erturk et al.^11^ conducted an observational, retrospective study evaluating the relationship between myocardial scar by ce-MRI and several electrocardiographic parameters of ventricular repolarization in patients with HFrEF. The authors observed prolonged measurements of QTc interval, Tp-e interval, and frontal QRS-T angle in patients with HFrEF who exhibited myocardial scar on ceMRI compared to those who did not.

Based on multivariable analysis, Tp-e interval was the best parameter to predict the presence of myocardial scar. The authors concluded that it is possible to predict myocardial scar in HFrEF using ECG parameters.^11^

There are limited reports concerning the relationship between the presence of myocardial scar and changes in the ECG. In experimental models, the induction of subendocardial scar formation has been shown to prolong repolarization time of the M cells,^9 , 12^ which determines the QT interval. Previous studies have shown a strong association between the presence of subendocardial scar and the abnormal ventricular repolarization in patients with coronary artery disease.^13^ Interestingly, community-based cardiac MRI studies have also demonstrated that the presence of subclinical scar detected by ceMRI was associated with increased mortality.^14^ Therefore, abnormal ventricular repolarization detected by ECG may be useful in predicting subclinical myocardial scar and identifying high risk patients.

Although the study presents encouraging results that add important information to prior reports,^11 , 13 , 15^ it has several limitations, including the small sample size, single-center patient selection and the retrospective and observational design. Large prospective studies are needed to validate the ventricular repolarization parameters proposed here. The investigators established a solid basis of information that will move forward the electrocardiography field in future studies.

This study, Erturk et al.^11^ also highlights the intimate relationship between myocardial fibrosis and disturbances in electrical conduction. There has been a substantial effort in understanding scar composition and related electrical instability.^3 , 5 , 7^ It is well known that areas of scarred tissue exhibit regions of slow conduction that favor reentry circuits and predispose to ventricular tachycardia. However, the underlying mechanism that yield ventricular fibrillation is less clear. Electrical instability is rarely the consequence of a single abnormality but rather a dynamic process involving of a group of factors that together lead to a fatal event. Consequently, new methods incorporating different modalities are needed to characterize ventricular electrical instability. The current study motivates the development of larger, prospective study to evaluate the use of ECG for screening of myocardial scar and consequent evaluation of ventricular arrhythmia risk.
